# Association between Neutrophil Percentage-to-Albumin Ratio and All-Cause Mortality in Critically Ill Patients with Coronary Artery Disease

**DOI:** 10.1155/2020/8137576

**Published:** 2020-08-31

**Authors:** Tienan Sun, Hua Shen, Qianyun Guo, Jiaqi Yang, Guangyao Zhai, Jingrui Zhang, Biyang Zhang, Yaodong Ding, Chenghui Cai, Yujie Zhou

**Affiliations:** ^1^Beijing Anzhen Hospital Affiliated to Capital Medical University, Beijing, China; ^2^Beijing Institute of Heart, Lung and Blood Vessel Disease, Beijing, China

## Abstract

**Background:**

Neutrophil percentage-to-albumin ratio (NPAR) has been proved to be associated with clinical outcome of many diseases. This study was aimed at exploring the independent effect of NPAR on all-cause mortality of critically ill patients with coronary artery disease (CAD).

**Method:**

NPAR was calculated as neutrophil percentage numerator divided by serum albumin concentration. Clinical endpoints were 30-day, 90-day, and 365-day all-cause mortality. Multivariable Cox proportional hazard models were performed to confirm the association between NPAR and all-cause mortality.

**Result:**

3106 patients with CAD were enrolled. All-cause mortality rates of 30 days (*P* < 0.001), 90 days (*P* < 0.001), and 365 days (*P* < 0.001) increased as NPAR tertiles increased. And after adjusting for possible confounding variables, NPAR was still independently associated with 30-day (third tertile group versus first tertile group: HR, 95% CI: 1.924, 1.471-2.516; *P* for trend < 0.001), 90-day (third tertile group versus first tertile group: HR, 95% CI: 2.053, 1.646-2.560; *P* for trend < 0.001), and 365-day (third tertile group versus first tertile group: HR, 95% CI: 2.063, 1.717-2.480; *P* for trend < 0.001) all-cause mortality in patients with CAD. Subgroup analysis did not find obvious interaction in most subgroups.

**Conclusion:**

NPAR was independently correlated with 30-day, 60-day, and 365-day all-cause mortality in critically ill patients with CAD.

## 1. Introduction

Although tremendous advances have been made in clinical and basic cardiovascular research over the past few decades, CAD still remains the major cause of death all over the world [1, 2]; readily accessible and inexpensive prognostic predictors are still necessary for patients with CAD, especially for critically ill patients.

Inflammation was proved to be related to atherosclerosis closely and played a key role in coronary plaque progression and adverse events after stent implantation [3–5]. Neutrophil, as one of classic cellular effectors, plays an important role in mediating inflammatory responses [3, 4]. Serum albumin concentration, as a classic measure of state of nutrition, is also effected by inflammation [6]. Lower serum albumin concentration was also proved to have a close connection to bad clinical outcomes in patients with CAD mainly due to malnutrition and inflammation [7, 8]. The pathophysiological changes caused by low serum albumin concentration also contributed to the development of cardiovascular diseases.

As a combination of two classical clinical evaluation parameters, NPAR is calculated as neutrophil percentage numerator divided by serum albumin concentration; a higher NPAR can indicate an increase in neutrophil percentage and a decrease in serum albumin concentration. Moreover, by the calculation of NPAR, the changes of these two indicators are amplified; especially in some cases, clinicians often ignore the significance of these two indicators, for example, when the neutrophil ratio is high and the albumin is low, but both are within the normal range. Previous studies also showed that a higher NPAR was associated with clinical outcomes of many diseases such as severe sepsis and acute kidney injury [9, 10]. In patients with ST-segment elevation myocardial infarction (STEMI), a higher NPAR was related to higher rates of death and reinfarction during hospitalization [11]. Based on the above evidence, we deduced that NPAR could influence the mortality of critically ill patients with CAD, and for all we know, there is no study reporting the influence of NPAR on mortality of critically ill patients with CAD.

## 2. Method

### 2.1. Data Source

We retrieved all data from an openly available critical care database named Medical Information Mart for Intensive Care III (MIMIC-III, version 1.4) [12], which included more than 60000 intensive care unit (ICU) stays and more than 50000 stays for adult patients. The data in MIMIC-III were collected from June 2001 to October 2012 in Beth Israel Deaconess Medical Center, including general information (patient demographics, birth and death, ICU admission, and discharge information), vital signs, laboratory data, the balance of body fluid, reports, medication, and nursing record. Protecting Human Research Participants exam was passed to gain access to the MIMIC-III database, and our certificate number is 9027152.

### 2.2. Study Population and Definition of NPAR

The selected population must meet the following requirements: (1) adult patients (age ≥ 18) who were diagnosed with coronary atherosclerosis or myocardial infarction according to ICD-9 diagnosis code and (2) parameters of neutrophil percent and serum albumin concentration were available after admission to ICU. Patients meeting the following criteria were excluded: (1) patients were under 18 years old and (2) lacking neutrophil percentage or serum albumin concentration data during their stay in the intensive care unit. NPAR was calculated as neutrophil percentage numerator divided by serum albumin concentration. Neutrophil percentage and serum albumin concentration were obtained by the first blood test after admission to the ICU and measured at the same time.

### 2.3. Data Extraction

Structure query language (SQL) was applied to collect patient information. Demographics, comorbidities and medical history, laboratory parameters, medication, scoring system, vital signs, and survival information were extracted. Demographics included age, gender, and ethnicity. Vital signs included heart beat (HR), systolic blood pressure (SBP), diastolic blood pressure (DBP), and mean blood pressure (MBP). Comorbidities and medical history included atrial fibrillation (AF), hypertension, chronic obstructive pulmonary disease (COPD), diabetes, hypercholesterolemia, congestive heart failure (CHF), prior myocardial infarction (prior MI), and prior stroke. Laboratory parameters included neutrophil, albumin, creatinine, activated partial thromboplastin time (APTT), white blood cell (WBC), hemoglobin, hematocrit, platelet, glucose, low-density lipoprotein cholesterol (LDL-C), troponin T, blood urea nitrogen (BUN), sodium, potassium, high-density lipoprotein cholesterol (HDL-C), international normalization ratio (INR), total cholesterol (TC), alanine aminotransferase (ALT), prothrombin time (PT), and C-reactive protein (CRP). Medication included beta-blockers, aspirin, thienopyridines, oral anticoagulants, angiotensin-converting enzyme inhibitors (ACEIs), angiotensin receptor blockers (ARBs), and statins. Scoring system included sequential organ failure assessment score (SOFA) [13] and simplified acute physiology score II (SAPS II) [14]. All the laboratory parameters were collected within 48 hours after admission to the ICU. Vital signs were extracted from a table named “vitalsfirstday” of the MIMIC-III database. Comorbidities and medical history were extracted from a table named “diagnoses_icd” of the MIMIC-III database. Laboratory parameters were extracted from a table named “labevents” of the MIMIC-III database. Medication use was extracted from a table named “prescriptions” of the MIMIC-III database. SOFA and SAPS II were extracted from a table named “sofa” and “sapsii” of the MIMIC-III database.

The endpoints of the study were 30-day, 90-day, and 365-day all-cause mortality. Survival information was extracted from a table named “patients” of the MIMIC-III database.

### 2.4. Statistical Analysis

All the patients with CAD were stratified according to NPAR tertiles. All continuous variables in this study were nonnormally distributed, and they were presented as median and interquartile range (IQR). Categorical data was summarized as number and percentage. Kruskal–Wallis or chi-square test was performed to evaluate statistical differences among different groups of NPAR.

Survival rates of different groups were compared by Log-rank tests, and the Kaplan–Meier curves were built. Multivariable Cox proportional hazard models were developed to evaluate the independent effect of NAPR on 30-day, 90-day, and 365-day all-cause mortality. The first tertile group of NPAR served as the reference group, and the results were summarized as hazard ratios (HRs) with 95% confidence intervals (CIs). Variables with *P* < 0.05 in the univariate analysis and cardiovascular risk factors were included into the multivariate Cox proportional hazard models. In model I, age, gender, and ethnicity were incorporated into adjustment. In model II, age, ethnicity, gender, length of ICU stay (ICU LOS), body mass index (BMI), SBP, DBP, HR, AMI, diabetes, hypercholesterolemia, prior MI, CHF, AF, hypertension, beta-blockers, oral anticoagulants, ACEIs, ARBs, BUN, glucose, platelet, WBC, ALT, INR, LDL-C, HDL-C, TC, troponin T, CRP, SOFA, and SAPS II were incorporated into the model. Subgroup analysis was used to determine the influence of NPAR on 30-day all-cause mortality in different subgroups, and *P* for interaction was calculated.

Receiver operating characteristic (ROC) curve was applied to evaluate the sensitivity and specificity of NPAR. DeLong test was applied to compare the area under the curves (AUC) of different parameters.

A *P* value of <0.05 was considered to be statistically significant. MedCalc and SPSS 23.0 (IBM Corporation) were used to conduct statistical analysis. GraphPad Prism was used to draw Kaplan–Meier curves and ROC curves.

## 3. Result

### 3.1. Patient Characteristics

**Figure 1:**
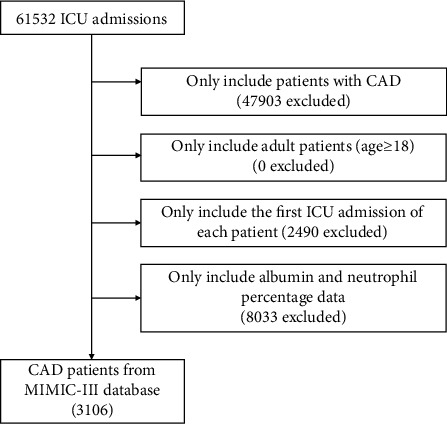
Flow chart of study population. ICU: intensive care unit; CAD: coronary artery disease.

3106 critically ill patients with CAD were enrolled in this study (Figure 1), whose characteristics stratified by NPAR tertiles were recorded. 1023 patients were included in the first tertile group (NPAR < 22.1), 1058 patients were included in second tertile group (22.1 ≤ NPAR < 27.9), and 1025 patients were included in third tertile group (NPAR ≥ 27.9). As displayed in Table 1, the median (IQR) of the NPAR level was 24.7 (20.8-30.0). 1975 men and 1131 women were included, most of whom were white. Patients in the highest tertile of the NPAR level presented more comorbidities or history of hypercholesterolemia, CHF, AF, COPD, prior MI, and prior stroke and less comorbidities of hypertension and diabetes. Moreover, patients in the highest tertile of the NPAR level received less thienopyridines, oral anticoagulants, beta-blockers, ACEIs, ARBs, and statin treatment. They also had higher HR, WBC, platelet, creatinine, BUN, sodium, PT, APTT, INR, CRP, SOFA, and SAPS II but lower BMI, SBP, DBP, MBP, hemoglobin, LDL-C, HDL-C, and TC.

### 3.2. NPAR and Outcomes

As shown in Table 2, the overall in-hospital, 30-day, 90-day, and 365-day all-cause mortality was 15.9%, 17.9%, 25.5%, and 34.6%, respectively. The rates of in-hospital, 30-day, 90-day, and 365-day all-cause mortality were all significantly increased as the NPAR tertiles increased. Moreover, ICU LOS significantly increased in the higher NPAR group.

**Figure 2:**
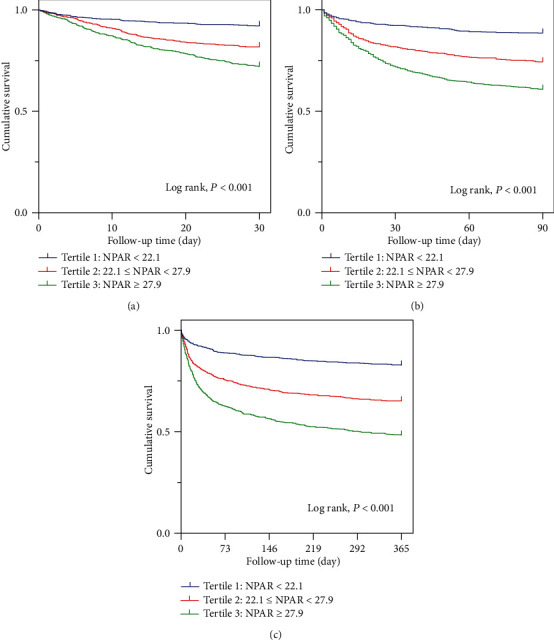
(a) Kaplan-Meier curves showing the association between the NPAR tertiles and 30-day all-cause mortality. (b) Kaplan-Meier curves showing the association between the NPAR tertiles and 90-day all-cause mortality. (c) Kaplan-Meier curves showing the association between the NPAR tertiles and 365-day all-cause mortality.

The survival curves of 30-day (Log rank, *P* < 0.001), 90-day (Log rank, *P* < 0.001), and 365-day (Log rank, *P* < 0.001) all-cause mortality stratified by the tertiles of NPAR are shown in Figure 2, which demonstrated significantly lower cumulative survivals with higher NPAR tertiles. The independent effect of NAPR on all-cause mortality was confirmed by Cox regression models. In model I, age, ethnicity, and gender, were incorporated into the regression model; compared with the first tertile, the highest 30-day (third tertile group versus first tertile group: HR, 95% CI: 3.387, 2.635-4.353; *P* for trend < 0.001), 90-day (third tertile group versus first tertile group: HR, 95% CI: 3.485, 2.831-4.290; *P* for trend < 0.001), and 365-day (third tertile group versus first tertile group: HR, 95% CI: 3.315, 2.789-3.940; *P* for trend < 0.001) all-cause mortality was confirmed in the highest tertile of NPAR. When examined as continuous variables in model I, each unit's higher NPAR was associated with increased 30-day (HR, 95% CI: 1.051, 1.042-1.060; *P* < 0.001), 90-day (HR, 95% CI: 1.054, 1.046-1.061; *P* < 0.001), and 365-day (HR, 95% CI: 1.053, 1.046-1.060; *P* < 0.001) all-cause mortality. In model II, age, ethnicity, gender, length of ICU stay (ICU LOS), body mass index (BMI), SBP, DBP, HR, AMI, diabetes, hypercholesterolemia, prior MI, CHF, AF, hypertension, beta-blockers, oral anticoagulants, ACEIs, ARBs, BUN, glucose, platelet, WBC, ALT, INR, LDL-C, HDL-C, TC, troponin T, CRP, SOFA, and SAPS II were incorporated. NPAR was still independently associated with 30-day (third tertile group versus first tertile group: HR, 95% CI: 1.924, 1.471-2.516; *P* for trend < 0.001), 90-day (third tertile group versus first tertile group: HR, 95% CI: 2.053, 1.646-2.560; *P* for trend < 0.001), and 365-day (third tertile group versus first tertile group: HR, 95% CI: 2.063, 1.717-2.480; *P* for trend < 0.001) all-cause mortality in patients with CAD. When examined as continuous variables in model II, each unit's higher NPAR was still associated with increased 30-day (HR, 95% CI: 1.032, 1.021-1.043; *P* < 0.001), 90-day (HR, 95% CI: 1.032, 1.023-1.040; *P* < 0.001), and 365-day (HR, 95% CI: 1.032, 1.024-1.040; *P* < 0.001) all-cause mortality independently (Table 3).

**Figure 3:**
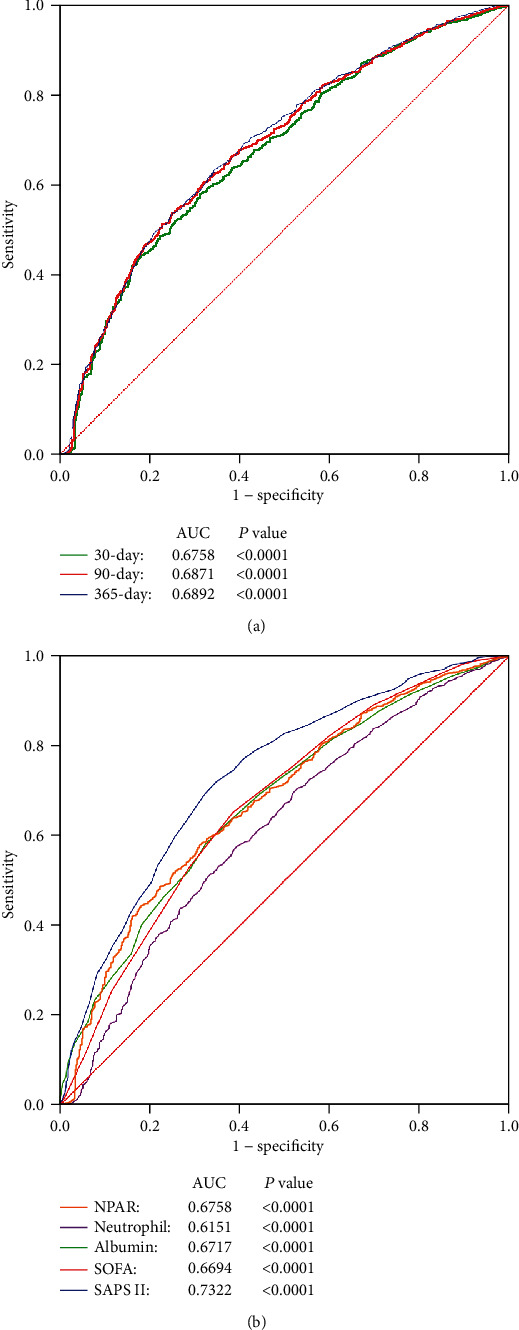
(a) ROC curves for the prediction of 30-day, 90-day, and 365-day all-cause mortality. (b) ROC curves for the prediction of 30-day all-cause mortality of NPAR, neutrophil percentage, serum albumin concentration, SOFA score, and SAPS II score.

The ability to predict all-cause mortality of NPAR is presented in Figure 3. The AUCs of NPAR for 30-day, 90-day, and 365-day mortality were 0.6758, 0.6871, and 0.6892, respectively. Comparing AUCs, the ability to predict the 30-day mortality of NPAR was proved to be lower than that of SAPS II (*P* = 0.009) but better than that of neutrophil percentage (*P* < 0.0001) alone. Comparing with the SOFA score (*P* = 0.7254) and albumin (*P* = 0.6521), no significant difference was found.

### 3.3. Subgroup Analysis

In most subgroups, no obvious interaction was observed. But increased risk of 30-day all-cause mortality was observed in patients with SAPS II score < 39 and BUN < 25 (*P* for interaction = 0.002, 0.003) (Table 4).

## 4. Discussion

This study showed that NPAR was an independent risk factor of 30-day, 90-day, and 365-day all-cause mortality in patients with CAD, even after adjusting for possible confounding variables. ROC curves revealed that NPAR had a moderate ability to predict all-cause mortality of critically ill patients with CAD. From the subgroup analysis, we did not find obvious interaction in most subgroups.

Inflammation was associated with atherosclerosis closely and played an important role in coronary plaque progression and adverse events after stent implantation [3–5]. A previous study showed that reducing classical inflammatory cascade contributed to reducing CAD-related adverse events [15]. WBC played a vital part in the host's defense against damage. In patients with stable angina pectoris or acute coronary syndrome, increased white blood cells were associated with poorer prognosis [16, 17]. Neutrophil, as an important component of WBC and one of the classic cellular effectors, played an important role in mediating inflammatory responses [3, 4]. Previous studies also showed that high neutrophil-lymphocyte ratio was related to cardiovascular mortality during hospitalization and poor outcomes in patients with STEMI [18, 19]. Besides, high neutrophil-lymphocyte ratio may contribute to coronary thrombus in patients with non-ST-element elevation acute myocardial infarction [20].

As a marker of nutritional condition and principal component of colloid osmotic pressure, serum albumin concentration was also effected by inflammation [6]. Previous studies showed that low serum albumin concentration was a strong prognostic marker for many diseases, mainly due to malnutrition and inflammation [6, 21]. Low serum albumin concentration was proved to be related to the development of ischemic heart disease and was proved to be an independent predictor of first or recurrent myocardial infarction (MI) [22–24]. Moreover, lower serum albumin concentration was also proved to be connected with worse clinical outcomes in patients with CAD [7, 8]. For patients with STEMI, low serum albumin levels, even within the normal range, could still influence long-term all-cause mortality [7]. A study that enrolled 1316 patients with CAD revealed that decreased albumin could predict a higher rate of all-cause death after percutaneous transluminal coronary intervention [8]. And a previous study also explained that why low albumin concentration could affect the outcome of patients with CAD, myocardial edema, and fluid retention played a key part in the progress of disease [23].

As a combination of two classical clinical evaluation parameters, NPAR was proved to be an independent predictor for clinical outcomes of many diseases such as severe sepsis, acute kidney injury, and STEMI [9–11], which had the advantage of simplicity, cheapness, and timeliness. A recent study demonstrated that a higher NPAR was related to higher rates of death and reinfarction during hospitalization in patients with STEMI [11]. Our study draw a similar conclusion that NPAR was an independent marker for all-cause mortality and had a moderate ability to predict all-cause mortality of critically ill patients with CAD. Although both neutrophil percentage and albumin could influence the outcomes of patients with CAD, NPAR may offer more predictive power from ROC curves. Moreover, comparing AUCs between NPAR and SOFA score, no significant difference was observed; although it was rash to think that NPAR was as effective as SOFA, at least this reminded us that when SOFA score cannot be calculated in some special circumstances, NPAR may be able to provide guidance for our clinical work. ROC curves also showed that the ability to predict 30-day mortality of NPAR was proved to be lower than that of SAPS II; as mentioned above, it was unrealistic for NPAR to achieve the same effectiveness as traditional classic scores, but considering the complexity of the calculation of SAPS II, NPAR had certain advantages in terms of simplicity. Given the low cost, availability, and capacity to predict mortality, NPAR is clinically valuable for critically ill patients with CAD. Especially in some special cases, such as remote areas with underdeveloped medical services or when patients are unable to perform more complex tests, NPAR may alert the clinicians.

## 5. Limitation

This study was a single retrospective study; inevitable bias may affect the authenticity of the results. In general, the more key variables a model contains, the more accurate its predictions will be. But constrained by public database, a lot of information that may affect the model was not collected, like smoking and alcohol. Moreover, neutrophil percentage and serum albumin concentration used in this analysis were obtained by the first blood test after admission to the ICU, but given the dynamic nature of these indicators, random errors caused by using only the first blood results were inevitable. The inability to dynamically observe NPAR was also one of the flaws of this study. In addition to this, other important information was also not collected such as specific causes of death, specific coronary artery lesions, type of myocardial infarction, specific clinical symptom, and left ventricular ejection fraction. Moreover, due to lack of albumin data, the sample size of this study declined significantly. In order to verify the conclusion of this study, a prospective case-control study may be needed.

## 6. Conclusions

NPAR was independently correlated with 30-day, 90-day, and 365-day all-cause mortality in critically ill patients with CAD. A prospective case-control study was needed to verify this conclusion.

## Figures and Tables

**Table 1:** Characteristics of study patients by NPAR tertiles.

Characteristics	Total (*n* = 3106)	Tertiles of NPAR			*P* value
		Tertile 1 (*n* = 1023) <22.1	Tertile 2 (*n* = 1058) ≥22.1, <27.9	Tertile 3 (*n* = 1025) ≥27.9	
Age (years)	72.5 (63.6-81.0)	69.0 (60.3-78.0)	73.6 (63.9-81.9)	75.0 (66.6-82.6)	<0.001
Gender, *n* (%)					<0.001
Male	1975 (63.6)	723 (70.7)	644 (60.9)	608 (59.3)	
Female	1131 (36.4)	300 (29.3)	414 (39.1)	417 (40.7)	
Ethnicity, *n* (%)					0.450
White	2241 (72.2)	727 (71.1)	783 (74.0)	731 (71.3)	
Black	160 (5.2)	50 (4.9)	55 (5.2)	55 (5.4)	
Other	705 (22.6)	246 (24)	220 (20.8)	239 (23.3)	
BMI (kg/m^2^)	27.4 (23.8-31.1)	28.2 (24.9-31.8)	27.3 (23.6-31.2)	26.6 (23.0-30.1)	<0.001
HR (beats/minute)	84 (74-93)	83 (75-91)	83 (73-92)	86 (75-96)	<0.001
SBP (mmHg)	113 (104-123)	114 (106-124)	113 (105-124)	112 (102-122)	<0.001
DBP (mmHg)	56 (51-63)	58 (53-64)	57 (51-64)	55 (50-62)	<0.001
MBP (mmHg)	74 (69-81)	75 (71-81)	74 (69-81)	73 (67-80)	<0.001
AMI, *n* (%)	93 (3.0)	19 (1.9)	32 (3.0)	42 (4.1)	0.012
Comorbidities and medical history, *n* (%)					
Hypertension	1476 (47.5)	596 (58.3)	478 (45.2)	402 (39.2)	<0.001
Diabetes	1220 (39.3)	406 (39.7)	450 (42.5)	364 (35.5)	0.004
Hypercholesterolemia	1258 (40.5)	521 (50.9)	407 (38.5)	330 (32.2)	<0.001
CHF	1576 (50.7)	420 (41.1)	615 (58.1)	541 (52.8)	<0.001
AF	1273 (41.0)	386 (37.7)	445 (42.1)	442 (43.1)	0.031
COPD	93 (3.0)	19 (1.9)	32 (3.0)	42 (4.1)	0.012
Prior MI	493 (15.9)	136 (13.3)	180 (17.0)	177 (17.3)	0.022
Prior stroke	95 (3.1)	22 (2.2)	37 (3.5)	36 (3.5)	0.120
Laboratory parameters					
Neutrophil (%)	80.7 (72.0-87.0)	70.3 (63.0-77.2)	82.1 (76.0-87.0)	86.5 (81.1-95.0)	<0.001
Albumin (g/dL)	3.2 (2.7-3.7)	3.8 (3.5-4.0)	3.3 (3.0-3.5)	2.5 (2.3-2.8)	<0.001
WBC (10^9^/L)	10.0 (7.5-13.0)	9.3 (7.0-12.1)	10.0 (7.6-13.0)	10.9 (7.9-13.9)	<0.001
Hemoglobin (g/dL)	10.3 (9.3-11.5)	10.6 (9.5-11.9)	10.4 (9.4-11.5)	10.0 (9.1-10.9)	<0.001
Hematocrit (%)	30.7 (27.8-34.0)	31.0 (27.9-34.9)	31.0 (28.2-34.2)	31.0 (27.4-32.7)	<0.001
Platelet (10^9^/L)	208.0 (149.0-289.0)	195.0 (146.0-262.0)	207.0 (151.0-288.0)	228.0 (151.0-316.0)	<0.001
Glucose (mg/dL)	123.0 (100.0-155.0)	122.0 (101.0-151.0)	125.0 (102.0-159.0)	121.0 (98.0-155.0)	0.077
LDL-C (mg/dL)	78.0 (57.0-107.0)	86.0 (64.0-117.0)	77.0 (57.0-103.0)	68.0 (50.0-97.5)	<0.001
HDL-C (mg/dL)	42.0 (33.0-51.0)	43.0 (36-51)	42.0 (33.0-52.0)	39.0 (30.0, 51.0)	0.001
TC (mg/dL)	148.0 (118.0-181.0)	160.0 (130.0-192.0)	145.0 (118.0-177.0)	134.0 (107.0-169.0)	<0.001
Troponin T (ng/mL)	0.09 (0.02-0.45)	0.07 (0.01-0.39)	0.1 (0.02-0.53)	0.09 (0.02-0.42)	0.001
Creatinine (mEq/L)	1.1 (0.8-1.7)	1.0 (0.8-1.4)	1.1 (0.8-1.9)	1.2 (0.8-2.1)	<0.001
BUN (mg/dL)	25.0 (17.0-40.0)	21.0 (15.0-30.0)	26.0 (18.0-42.0)	29 (19.0-48.0)	<0.001
Sodium (mmol/L)	138.0 (135.0-141.0)	138.0 (136.0-140.0)	138.0 (135.0-141.0)	139.0 (135.0-142.0)	<0.001
Potassium (mmol/L)	4.1 (3.8-4.5)	4.2 (3.9-4.4)	4.2 (3.8-4.4)	4.2 (3.8-4.4)	0.469
PT (seconds)	13.6 (12.7-15.2)	13.3 (12.5-14.3)	13.8 (12.8-15.6)	13.9 (12.9-15.6)	<0.001
APTT (seconds)	29.8 (26.2-38.9)	29.0 (25.8-37.7)	30.4 (26.4-39.4)	30.0 (26.4-39.4)	0.007
INR	1.2 (1.1-1.4)	1.1 (1.1-1.3)	1.2 (1.1-1.5)	1.2 (1.1-1.5)	<0.001
ALT (U/L)	26 (17-40)	25 (17-40)	27 (17-42)	29 (16-40)	0.140
CRP	13.0 (7.3-23.0)	9.4 (5.2-18.4)	11.0 (7.0-17.0)	19.5 (12.4-29.7)	<0.001
Medication use					
Aspirin	2911 (93.7)	957 (93.5)	991 (93.7)	963 (94.0)	0.928
Thienopyridines	1187 (38.2)	399 (39.0)	445 (42.1)	343 (33.5)	<0.001
Oral anticoagulants	842 (27.1)	283 (27.7)	322 (30.4)	237 (23.1)	0.001
Beta-blockers	2434 (78.4)	886 (84.7)	827 (78.2)	741 (72.3)	<0.001
ACEIs	1439 (46.3)	545 (53.3)	492 (46.5)	402 (39.2)	<0.001
ARBs	238 (7.7)	89 (8.7)	95 (9.0)	54 (5.3)	0.002
Statins	2013 (67.7)	803 (78.5)	725 (68.5)	575 (56.1)	<0.001
Scoring systems					
SOFA	5 (3-7)	4 (2-6)	4 (3-7)	5 (3-8)	<0.001
SAPS II	39.0 (31.0-49.0)	36.0 (29.0-44.5)	40.0 (32.0-47.0)	43.0 (34.0-52.0)	<0.001
NPAR	24.7 (20.8-30.0)	19.1 (16.8-20.7)	24.7 (23.2-26.2)	32.9 (30.0-37.4)	<0.001

Data are described as count (percentage) for categorical variables and median (interquartile range) for continuous variables. BMI: body mass index; HR: heart beat; SBP: systolic blood pressure; DBP: diastolic blood pressure; MBP: mean blood pressure; AMI: acute myocardial infarction; CHF: chronic heart failure; AF: atrial fibrillation; COPD: chronic obstructive pulmonary disease; prior MI: prior myocardial infarction; WBC: white blood cell; LDL-C: low-density lipoprotein cholesterol; HDL-C: high-density lipoprotein cholesterol; TC: total cholesterol; BUN: blood urea nitrogen; PT: prothrombin time; APTT: activated partial thromboplastin time; INR: international normalization ratio; ALT: alanine aminotransferase; CRP: C-reactive protein; ACEIs: angiotensin-converting enzyme inhibitors; ARBs: angiotensin receptor blockers; SOFA: sequential organ failure assessment score [13]; SAPS II: simplified acute physiology score II [14].

**Table 2:** Outcome of the study patients by NPAR tertiles.

Outcomes	Total (*n* = 3106)	Tertiles of NPAR			*P* value
		Tertile 1 (*n* = 1023) <22.1	Tertile 2 (*n* = 1058) ≥22.1, <27.9	Tertile 3 (*n* = 1025) ≥27.9	
Mortality, *n* (%)					
30-day mortality	577 (17.9)	80 (7.8)	192 (18.1)	285 (27.8)	<0.001
90-day mortality	792 (25.5)	118 (11.5)	272 (25.7)	402 (39.2)	<0.001
365-day mortality	1074 (34.6)	176 (17.2)	369 (34.9)	529 (51.6)	<0.001
In-hospital	495 (15.9)	71 (6.9)	175 (16.5)	249 (24.3)	<0.001
ICU LOS (days)	3.29 (1.83-7.32)	2.46 (1.30-4.33)	3.26 (1.83-6.96)	5.08 (2.24-11.13)	<0.001

Data are expressed as count (percentage) for categorical variables and median (interquartile range) for continuous variables. ICU LOS: length of ICU stay.

**Table 3:** The association between all-cause mortality and NPAR.

	Nonadjusted			Model 1			Model 2		
	HR (95% CIs)	*P*	*P* for trend	HR (95% CIs)	*P*	*P* for trend	HR (95% CIs)	*P*	*P* for trend
30-day all-cause mortality									
Tertile 1 (NPAR < 22.1)	1.0 (ref)		<0.001	1.0 (ref)		<0.001	1.0 (ref)		<0.001
Tertile 2 (22.1 ≤ NPAR < 27.9)	2.449 (1.887, 3.179)	<0.001		2.185 (1.680, 2.842)	<0.001		1.589 (1.212, 2.084)	0.001	
Tertile 3 (NPAR ≥ 27.9)	3.929 (3.066, 5.035)	<0.001		3.387 (2.635, 4.353)	<0.001		1.924 (1.471, 2.516)	<0.001	
Continuous	1.056 (1.048, 1.065)	<0.001		1.051 (1.042, 1.060)	<0.001		1.032 (1.021, 1.043)	<0.001	
90-day all-cause mortality									
Tertile 1 (NPAR < 22.1)	1.0 (ref)		<0.001	1.0 (ref)		<0.001	1.0 (ref)		<0.001
Tertile 2 (22.1 ≤ NPAR < 27.9)	2.422 (1.952, 3.007)	<0.001		2.177 (1.751, 2.707)	<0.001		1.701 (1.361, 2.126)	<0.001	
Tertile 3 (NPAR ≥ 27.9)	4.006 (3.262, 4.919)	<0.001		3.485 (2.831, 4.290)	<0.001		2.053 (1.646, 2.560)	<0.001	
Continuous	1.059 (1.051, 1.066)	<0.001		1.054 (1.046, 1.061)	<0.001		1.032 (1.023, 1.040)	<0.001	
365-day all-cause mortality									
Tertile 1 (NPAR < 22.1)	1.0 (ref)		<0.001	1.0 (ref)		<0.001	1.0 (ref)		<0.001
Tertile 2 (22.1 ≤ NPAR < 27.9)	2.286 (1.910, 2.735)	<0.001		2.039 (1.701, 2.444)	<0.001		1.629 (1.354, 1.960)	<0.001	
Tertile 3 (NPAR ≥ 27.9)	3.823 (3.223, 4.535)	<0.001		3.315 (2.789, 3.940)	<0.001		2.063 (1.717, 2.480)	<0.001	
Continuous	1.058 (1.052, 1.065)	<0.001		1.053 (1.046, 1.060)	<0.001		1.032 (1.024, 1.040)	<0.001	

HR: hazard ratio; CI: confidence interval. Models were derived from Cox proportional hazard regression models. Nonadjusted model, adjusted for none. Adjust I model, adjusted for age, ethnicity, and gender. Adjust II model, adjusted for age, ethnicity, gender, ICU LOS, BMI, SBP, DBP, HR, MI, DM, hypercholesterolemia, prior MI, congestive heart failure, atrial fibrillation, hypertension, beta-blockers, oral anticoagulant, ACEI, ARB, blood urea nitrogen, glucose, platelet, WBC, ALT, INR, LDL-C, HDL-C, TC, TnT, CRP, SOFA, and SAPS II.

**Table 4:** Subgroup analysis of associations between 30-day all-cause mortality and NPAR.

	*N*	NPAR < 22.1 (reference)	22.1 ≤ NPAR < 27.9 HR (95% CIs)	NPAR ≥ 27.9 HR (95% CIs)	*P* for interaction
AMI					0.886
No	3013	1.0 (ref)	2.480 (1.904, 3.230)	3.951 (3.072, 5.082)	
Yes	93	1.0 (ref)	1.450 (0.281, 7.477)	2.878 (0.644, 12.863)	
CHF					0.862
No	1530	1.0 (ref)	2.946 (1.956, 4.438)	4.618 (3.149, 6.771)	
Yes	1576	1.0 (ref)	1.899 (1.353, 2.666)	3.172 (2.290, 4.393)	
AF					0.912
No	1833	1.0 (ref)	2.069 (1.470, 2.912)	3.594 (2.610, 4.949)	
Yes	1273	1.0 (ref)	3.013 (2.000, 4.540)	4.398 (2.960, 6.535)	
Hypertension					0.066
No	1630	1.0 (ref)	2.197 (1.558, 3.099)	3.169 (2.281, 4.403)	
Yes	1476	1.0 (ref)	2.459 (1.641, 3.682)	4.528 (3.094, 6.629)	
Diabetes					0.934
No	1886	1.0 (ref)	2.585 (1.839, 3.632)	4.044 (2.936, 5.571)	
Yes	1220	1.0 (ref)	2.269 (1.511, 3.406)	3.757 (2.534, 5.568)	
Hypercholesterolemia					0.149
No	1848	1.0 (ref)	2.190 (1.572, 3.051)	3.324 (2.426, 4.555)	
Yes	1258	1.0 (ref)	2.635 (1.722, 4.032)	4.573 (3.043, 6.872)	
Prior MI					0.428
No	2613	1.0 (ref)	2.428 (1.822, 3.236)	4.020 (3.064, 5.276)	
Yes	493	1.0 (ref)	2.412 (1.288, 4.518)	3.371 (1.835, 6.190)	
Beta-blockers					0.700
No	672	1.0 (ref)	2.722 (1.639, 4.520)	3.715 (2.283, 6.046)	
Yes	2434	1.0 (ref)	2.183 (1.605, 2.968)	3.590 (2.682, 4.806)	
Oral anticoagulant					0.560
No	2264	1.0 (ref)	2.512 (1.895, 3.330)	3.744 (2.866, 4.890)	
Yes	842	1.0 (ref)	2.494 (1.250, 4.976)	4.468 (2.288, 8.725)	
ACEIs					0.907
No	1667	1.0 (ref)	2.142 (1.567, 2.927)	3.473 (2.590, 4.655)	
Yes	1439	1.0 (ref)	2.830 (1.761, 4.548)	3.649 (2.278, 5.845)	
ARBs					0.452
No	2868	1.0 (ref)	2.482 (1.903, 3.237)	3.811 (2.960, 4.907)	
Yes	238	1.0 (ref)	1.912 (0.478, 7.645)	5.299 (1.435, 19.577)	
BUN (mg/dL)					0.003
<25	1542	1.0 (ref)	3.535 (2.108, 5.929)	5.892 (3.579, 9.701)	
≥25	1564	1.0 (ref)	1.596 (1.180, 2.159)	2.358 (1.772, 3.139)	
Glucose (mg/dL)					0.485
<123	1540	1.0 (ref)	2.052 (1.387, 3.037)	3.443 (2.393, 4.954)	
≥123	1566	1.0 (ref)	2.767 (1.946, 3.934)	4.421 (3.149, 6.209)	
Platelet (10^9^/L)					0.858
<208	1539	1.0 (ref)	2.287 (1.641, 3.187)	3.990 (2.905, 5.480)	
≥208	1567	1.0 (ref)	2.824 (1.844, 4.327)	4.321 (2.880, 6.482)	
WBC (10^9^/L)					0.134
<10	1409	1.0 (ref)	2.010 (1.357, 2.976)	2.797 (1.898, 4.123)	
≥10	1697	1.0 (ref)	2.750 (1.931, 3.917)	4.426 (3.167, 6.185)	
ALT (U/L)					0.436
<26	1488	1.0 (ref)	2.647 (1.793, 3.909)	4.349 (3.001, 6.304)	
≥26	1618	1.0 (ref)	2.264 (1.593, 3.216)	3.545 (2.540, 4.949)	
INR					0.046
<1.2	1272	1.0 (ref)	2.639 (1.685, 4.133)	4.993 (3.289, 7.581)	
≥1.2	1834	1.0 (ref)	2.151 (1.559, 2.968)	3.178 (2.334, 4.328)	
LDL-C (mg/dL)					0.380
<84.1	1539	1.0 (ref)	2.059 (1.441, 2.942)	3.355 (2.402, 4.868)	
≥84.1	1567	1.0 (ref)	2.852 (1.946, 4.179)	4.365 (3.010, 6.330)	
HDL-C (mg/dL)					0.732
<43.5	1552	1.0 (ref)	2.312 (1.592, 3.356)	3.842 (2.711, 5.444)	
≥43.5	1554	1.0 (ref)	2.574 (1.787, 3.708)	3.915 (2.744, 5.585)	
TC (mg/dL)					0.452
<153.6	1517	1.0 (ref)	2.458 (1.672, 3.614)	3.663 (2.528, 5.307)	
≥153.6	1589	1.0 (ref)	2.457 (1.724, 3.503)	4.182 (2.995, 5.839)	
Troponin T (ng/mL)					0.644
<0.02	1446	1.0 (ref)	1.960 (1.297, 2.960)	3.731 (2.565, 5.427)	
≥0.02	1660	1.0 (ref)	2.622 (1.860, 3.697)	3.890 (2.789, 5.425)	
CRP (mg/L)					0.127
<13.0	1550	1.0 (ref)	2.791 (2.023, 3.849)	3.894 (2.736, 5.541)	
≥13.0	1556	1.0 (ref)	1.959 (1.254, 3.061)	4.053 (2.732, 6.014)	
SOFA					0.099
<5	1513	1.0 (ref)	2.262 (1.408, 3.634)	4.510 (2.882, 7.059)	
≥5	1593	1.0 (ref)	2.484 (1.817, 3.396)	3.288 (2.441, 4.430)	
SAPS II					0.002
<39	1472	1.0 (ref)	4.189 (2.310, 7.597)	7.064 (3.940, 12.662)	
≥39	1634	1.0 (ref)	1.789 (1.337, 2.395)	2.477 (1.883, 3.259)	
BMI (kg/m^2^)					0.297
<27.4	1564	1.0 (ref)	2.245 (1.578, 3.194)	3.412 (2.444, 4.764)	
≥27.4	1542	1.0 (ref)	2.579 (1.749, 3.802)	4.296 (2.959, 6.235)	
SBP (mmHg)					0.410
<113	1547	1.0 (ref)	2.421 (1.720, 3.408)	3.564 (2.574, 4.933)	
≥113	1559	1.0 (ref)	2.421 (1.617, 3.625)	4.206 (2.865, 6.174)	
DBP (mmHg)					0.465
<57	1561	1.0 (ref)	3.066 (2.053, 4.579)	4.711 (3.211, 6.913)	
≥57	1545	1.0 (ref)	2.201 (1.425, 2.866)	3.355 (2.404, 4.682)	
HR (beats/minute)					0.149
<84	1570	1.0 (ref)	3.182 (2.135, 4.743)	5.012 (3.398, 7.393)	
≥84	1536	1.0 (ref)	1.957 (1.382, 2.771)	3.155 (2.286, 4.356)	
ICU LOS (day)					0.004
<3.3	1554	1.0 (ref)	3.009 (2.060, 4.397)	5.284 (3.641, 7.671)	
≥3.3	1552	1.0 (ref)	1.832 (1.279, 2.624)	2.709 (1.937, 3.788)	

HR: hazard ratio; CI: confidence interval. AMI: acute myocardial infarction; CHF: chronic heart failure; AF: atrial fibrillation; Prior MI: prior myocardial infarction; ACEIs: angiotensin-converting enzyme inhibitors; ARBs: angiotensin receptor blockers; BUN: blood urea nitrogen; WBC: white blood cell; ALT: alanine aminotransferase; INR: international normalization ratio; LDL-C: low-density lipoprotein cholesterol; HDL-C: high-density lipoprotein cholesterol; TC: total cholesterol; CRP: C-reactive protein; SOFA: sequential organ failure assessment score; SAPS II: simplified acute physiology score II; BMI: body mass index; SBP: systolic blood pressure; DBP: diastolic blood pressure; HR: heart beat; ICU LOS: length of ICU stay.

## Data Availability

All data used in this analysis were from an openly available critical care database named MIMIC-III. Protecting Human Research Participants exam was passed to gain access to the MIMIC-III database, and our certificate number is 9027152.

## Supplementary Materials

Supplementary Materials 1. The file named “Original data” was the raw data for all the analysis of this article. 2. The file named “selected population icd9-code” was used to show all icd9_code related to the diagnosis of coronary artery disease.
