# Supplementary material for: Association between Neutrophil Percentage-to-Albumin Ratio and All-Cause Mortality in Critically Ill Patients with Coronary Artery Disease
Source: Biomed Res Int. 2020 Aug 31;2020:8137576. doi: 10.1155/2020/8137576 (PMC7479485; doi:10.1155/2020/8137576)
Supplement: Supplementary Materials — 1. The file named “Original data” was the raw data for all the analysis of this article. 2. The file named “selected population icd9-code” was used to show all icd9_code related to the diagnosis of coronary artery disease. [file 8137576.f1.zip › selected population icd9-code.docx]

ICD-9 diagnostic code containing the terms “coronary atherosclerosis of native coronary artery”, “chronic total occlusion of coronary artery”, “coronary atherosclerosis due to lipid rich plaque”, “coronary atherosclerosis due to calcified coronary lesion”, “acute myocardial infarction of anterolateral wall”, “acute myocardial infarction of anterior wall”, “acute myocardial infarction of inferolateral wall”, “acute myocardial infarction of inferoposterior wall”, “acute myocardial infarction of other inferior wall”, “acute myocardial infarction of other lateral wall”, “acute myocardial infarction of other specified sites”, “acute myocardial infarction of unspecified site,” or “old myocardial infarction”.
